# Recovery of Breakthrough Asthma Attacks Treated With Oral Steroids While on Monoclonal Antibody Therapy: Protocol for a Prospective Observational Study (BOOST)

**DOI:** 10.2196/46741

**Published:** 2023-06-23

**Authors:** Imran Howell, Mahdi Mahdi, Mona Bafadhel, Timothy S C Hinks, Sanjay Ramakrishnan, James Melhorn, Maisha Jabeen, Ian D Pavord

**Affiliations:** 1 Nuffield Department of Medicine University of Oxford Oxford United Kingdom; 2 Respiratory Medicine Unit National Institute for Health Research Respiratory Biomedical Research Centre Oxford United Kingdom; 3 King's College London London United Kingdom

**Keywords:** asthma, monoclonal antibodies, steroids, monoclonal antibody, oral steroid, asthma attack, quality of life, adult, inflammatory response, prednisolone treatment, treatment

## Abstract

**Background:**

Asthma attacks are a common and important problem. Someone experiences an asthma attack in the United Kingdom every 10 seconds. Asthma attacks cause coughing, wheezing, breathlessness, and chest tightness and are highly stressful for patients. They result in reduced quality of life, with days lost from work or school. Asthma attacks are treated with oral corticosteroids (OCSs), but these have many short- and long-term side effects. Asthma monoclonal antibodies (mAbs) have revolutionized the treatment of severe asthma by reducing asthma attacks and OCS burden by over 50%, but some people still experience attacks while on mAbs. The MEX study showed that residual asthma attacks are broadly eosinophilic (high fractional exhaled nitric oxide [FeNO]) or noneosinophilic (low FeNO), but it did not measure response to OCS treatment. There is an evidence gap in understanding the clinical and inflammatory responses that occur when using OCSs to treat residual asthma attacks in patients taking asthma mAbs.

**Objective:**

The primary objective is to compare the clinical recovery between high-FeNO and low-FeNO attacks after acute treatment with oral prednisolone among people established on long-term asthma mAb treatment. The exploratory objective is to compare the inflammatory response to acute treatment with oral prednisolone between high-FeNO and low-FeNO attacks.

**Methods:**

BOOST (Breakthrough Asthma Attacks Treated With Oral Steroids) is a single-center, prospective observational study of 60 adults established on long-term asthma mAb treatment who receive acute treatment with oral prednisolone (usual care) for an asthma attack. The primary outcome will be the proportion of treatment failure (the need to start oral prednisolone or antibiotics or an unscheduled health care visit for asthma, following an attack) at day 28. The secondary outcomes will be the change in forced expiratory volume in 1 second and the change in visual analogue scale symptom score between the stable state, attack, day 7, and day 28 visits. The exploratory outcomes include the changes in sputum, nasal, and blood inflammometry between the stable state, attack, day 7, and day 28 visits.

**Results:**

The last asthma attack visit is anticipated to occur in December 2023. Data analysis and publication will take place in 2024.

**Conclusions:**

We will test the hypothesis that there is a difference in the rate of recovery of clinical and inflammatory measures between high-FeNO and low-FeNO asthma attacks that occur in patients on mAb therapy. The study data will help power a future randomized placebo-controlled trial of prednisolone treatment for nonsevere attacks in patients treated with asthma mAbs and will provide important information on whether corticosteroid treatment should be FeNO-directed.

**International Registered Report Identifier (IRRID):**

DERR1-10.2196/46741

## Introduction

Asthma is a common and important problem. Clinically, asthma is characterized by variable symptoms of breathlessness, coughing, chest tightness, and wheezing. Asthma attacks are acute deteriorations in asthma symptoms and affect a person’s physical activity, work, personal life, and mental health [1]. The presence of raised type 2 airway inflammation increases the risk of asthma attacks and lung function decline. Blood eosinophils and fractional exhaled nitric oxide (FeNO) have emerged as key biomarkers of type 2 inflammation [2].

The cornerstone of asthma treatment is inhaled corticosteroids (ICSs), which reduce type 2 inflammation. However, 10% of adults experience severe asthma with frequent asthma attacks, despite ICS treatment [3]. These attacks are treated with 5- to 7-day bursts of high-dose oral corticosteroids (OCSs), based on trial data showing a 50% lower risk of relapse at 21 days in comparison to a placebo [4]. However, all of the randomized placebo-controlled trials of OCSs for asthma attacks were conducted in an era before the widespread use of ICSs, implying that the efficacy of OCSs in mild to moderate attacks may be overstated. Moreover, OCSs have a significant acute side effect burden, and their cumulative toxicity is serious [5].

Asthma monoclonal antibody (mAb) therapies have revolutionized the treatment of severe asthma. They target specific parts of the type 2 inflammatory pathway and have fewer side effects than OCSs. The key classes of mAbs are anti-immunoglobulin E (omalizumab), anti–interleukin (IL) 5 and anti-IL5R (mepolizumab, reslizumab, and benralizumab), anti-IL4Rα (dupilumab), and anti–thymic stromal lymphopoietin (tezepelumab) [6]. The randomized trials of these drugs versus a placebo demonstrated an over 50% reduction in asthma attacks requiring OCSs [7]. On average, people continue to experience 1 asthma attack per year. The current practice is for these asthma attacks to be treated with OCSs.

Patients effectively treated with asthma biologics generally exhibit reduced type 2 inflammation. Consequently, they are ideal patients for testing the hypothesis that OCSs are only useful in treating attacks with evidence of type 2 inflammation. This has already been explored in patients with chronic obstructive pulmonary disease (COPD) but not asthma. Two randomized controlled trials undertaken in patients with COPD showed that among the patients with low blood eosinophil levels at the time of a COPD attack, OCS treatment did not improve outcomes, when compared with a placebo, in terms of treatment failure [8,9].

The MEX study was the first prospective, multicenter, observational study to characterize asthma attacks in patients on mepolizumab [10]. It found that attacks were often clinically mild and heterogeneous in nature. Attacks were broadly defined as eosinophilic (high FeNO) or noneosinophilic (low FeNO). BenRex is a similar ongoing study characterizing attacks in patients treated with benralizumab [11]. Neither study was designed to examine recovery from an attack after treatment with OCSs.

The findings from the MEX study point to a possible biomarker-directed option for treating asthma attacks based on FeNO. Attacks among people on anti-IL5 treatment with raised FeNO may imply ongoing IL-4, IL-13, or alarmin activity, which may be OCS-responsive [12]. The noneosinophilic attacks were frequently positive for viruses or bacteria and had a higher C-reactive protein level, indicating that they were probably pathogen-driven. This process would not be expected to respond to OCSs.

There is a large evidence gap in understanding the clinical and inflammatory responses that occur when using OCSs to treat asthma attacks in patients treated with asthma mAbs. Our prospective observational study will examine these responses to help inform whether a future randomized placebo-controlled trial of prednisolone treatment for nonsevere attacks in patients treated with asthma mAbs should be FeNO-directed.

## Methods

### Hypothesis

In people established on anti-IL5, anti-IL5Rα, and anti-IL4Rα mAb treatment for asthma, high-FeNO asthma attacks will show more clinical and inflammatory responses to acute oral prednisolone treatment than low-FeNO asthma attacks.

### Objectives

The primary objective is to compare the clinical recovery between high-FeNO and low-FeNO attacks after acute treatment with oral prednisolone among people established on anti-IL5, anti-IL5Rα, and anti-IL4Rα treatment for asthma. The exploratory objective is to compare the inflammatory response to acute treatment with oral prednisolone between high-FeNO and low-FeNO attacks among people established on anti-IL5, anti-IL5Rα, and anti-IL4Rα treatment for asthma.

### Study Design

BOOST (Breakthrough Asthma Attacks Treated With Oral Steroids) is a single-center, prospective observational study of 60 adults established on long-term asthma mAb treatment who receive acute treatment with oral prednisolone (usual care) for an asthma attack.

### Participant Recruitment

All participants will be recruited from the Oxford Special Airways Clinic, which is based at the Oxford Centre for Respiratory Medicine at the John Radcliffe Hospital. This service provides specialist assessments for patients with complex airway diseases and has a large cohort of patients on asthma mAb treatment under regular review. The service also provides a rapid assessment clinic for the clinical review of patients who experience an asthma attack while on mAb treatment.

There are 2 routes for a participant to enroll into the study. Participants can be enrolled into a study pool at stable state. If they exacerbate, they can contact the department to initiate an attack visit to the rapid assessment clinic. Alternatively, participants may directly enter the study at the time of an attack if they have contacted the rapid assessment clinic for review but are not in the study pool. These participants will have a stable state visit at least 2 months after the attack date.

If a participant in the study pool has not experienced an asthma attack when the study’s attack recruitment target has been met, they will be invited for an optional additional visit at stable state to form a stable control group.

### Ethical Considerations

BOOST is a substudy of the Oxford Airways Study (Integrated Research Application System Project number: 234581; Oxfordshire Research Ethics Committee B reference number: 18/SC/0361). The Oxford Airways Study is an observational study that will measure clinical measures, biomarkers, inflammation, and mediators among participants with airway diseases at stable state and during an acute attack.

Participants will be consented to the Oxford Airways Study at their first study visit.

### Inclusion Criteria

The inclusion criteria are as follows: (1) people treated with an anti-IL5, anti-IL5Rα, and anti-IL4Rα mAb for asthma (mepolizumab, benralizumab, reslizumab, or dupilumab) under the Oxford Special Airways Clinic; (2) patients established on the same mAb treatment for at least 2 months; (3) male or female patients aged 18 years or older; and (4) patients who experience at least 1 asthma attack requiring oral prednisolone treatment since starting mAb treatment.

### Exclusion Criteria

The general exclusion criteria are as follows: (1) maintenance OCS treatment for asthma or any other condition, (2) people who are unwilling or unable to attend follow-up visits, (3) people who are pregnant or are planning pregnancy at the time of study entry, and (4) people on long-term systemic immunosuppressive treatment for a condition other than asthma.

At asthma attack visit, the following exclusion criteria will be assessed: (1) pneumonia on a chest radiograph at the time of an attack, (2) any treatment with OCSs in the preceding 1 month, and (3) the trial physician’s opinion that a person needs hospitalization at the time of assessment.

### Intervention at Asthma Attack Visit

A consultation will be arranged at the rapid assessment clinic if a participant feels that their asthma symptoms have deteriorated and they need treatment. The participant will be assessed by the study physician via medical history taking, clinical examination, and chest radiograph examination. This is in line with our usual practice. If a diagnosis of an asthma attack is made and the participant is eligible, they will start the study-specific measurements (Table 1).

**Figure 1:**
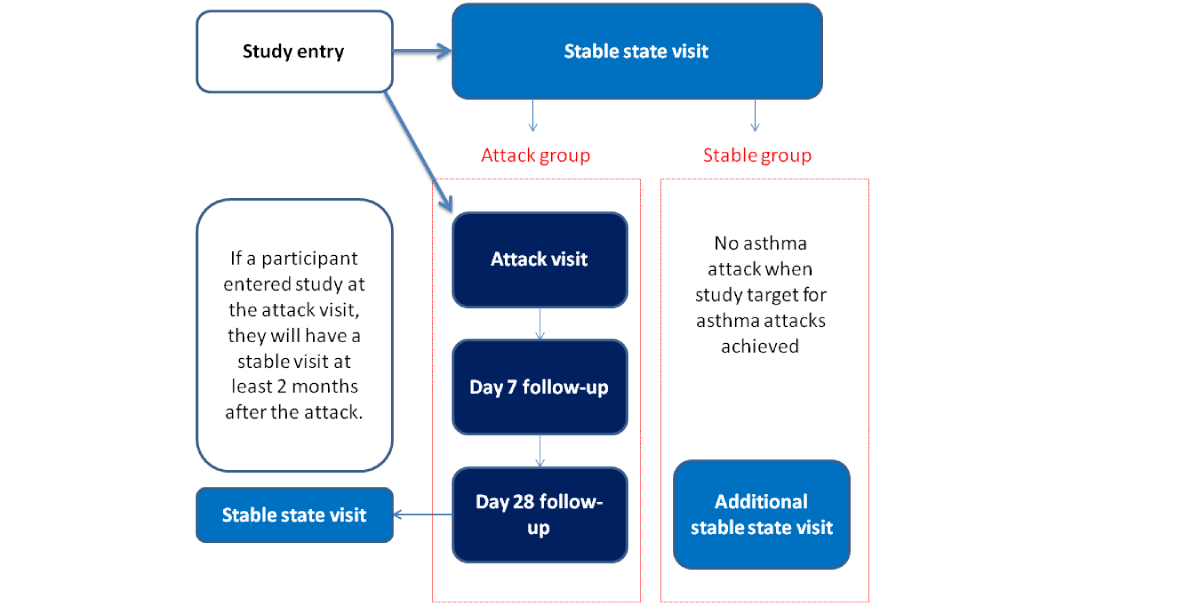
Summary of planned study visits.

Each participant will be treated with 40 mg of oral prednisolone once per day for 7 days as part of usual care for an asthma attack, as per British Thoracic Society guidelines. Antibiotic treatment will not be routinely prescribed at the asthma attack visit. If a participant is already on antibiotic therapy prior to the attack visit (eg, prescribed in general practice), this will be continued until the prescribed course is complete, as per usual care (Figure 1).

**Table 1:** Study measurements.

Measurements	Stable state visit	Asthma attack visit	Day 7 follow-up	Day 28 follow-up	Additional stable state visit
Informed consent	✓	✓			
Eligibility check	✓	✓			
Demographics	✓	✓			
Medical and asthma biologic history	✓	✓			
Medication check	✓	✓	✓	✓	✓
Physical examination	✓	✓	✓	✓	✓
Vital signs	✓	✓	✓	✓	✓
Spirometry	✓	✓	✓	✓	✓
Fractional exhaled nitric oxide	✓	✓	✓	✓	✓
Blood sampling	✓	✓	✓	✓	✓
Induced sputum sampling	✓	✓	✓	✓	✓
Nasosorption sampling	✓	✓	✓	✓	✓
Visual analogue scale symptoms	✓	✓	✓	✓	✓
Asthma Control Questionnaire and Asthma Control Test	✓	✓	✓	✓	✓
Medical notes review	✓	✓	✓	✓	✓
Chest x-ray		✓			
Nasal viral polymerase chain reaction test		✓			
Assessment for treatment failure			✓	✓	
Adverse event assessments			✓	✓	

### Unplanned Study Visits

If a participant feels that their asthma symptoms have deteriorated or that they need more treatment, between the attack visit and day 28 visit, they will be encouraged to contact the study team. As per usual care, the participant will be assessed by the study team, by the participant's general practitioner, or in an urgent care setting. If the participant is assessed by the study team, routine clinical measurements will be taken at the discretion of the study physician, but no research samples will be taken. If a participant is assessed by their general practitioner or in an urgent care setting, the study team will ask the participant what medical treatment they received and whether they needed a hospital visit. Where possible, this information will be corroborated with health records.

### Outcomes

The primary outcome is the comparison of the proportion of treatment failure at day 28 between high-FeNO and low-FeNO attacks.

The secondary outcomes are as follows: (1) comparison of the change in forced expiratory volume in 1 second (FEV_1_) from stable state to day 0, day 7, and day 28 between high-FeNO and low-FeNO attacks; (2) comparison of the change in visual analogue scale (VAS) symptom score from stable state to day 0, as well as daily VAS symptom score changes from day 0 to day 28, between high-FeNO and low-FeNO attacks; and (3) comparison of the change in Asthma Control Questionnaire and Asthma Control Test symptom scores from stable state to day 0, day 7, and day 28 between high-FeNO and low-FeNO attacks.

The exploratory outcomes are as follows: (1) comparison of the changes in sputum, blood, and nasal lining fluid inflammometry; (2) comparison of the changes in sputum and blood transcriptomics; (3) comparison of microbiology statuses at asthma attack; (4) comparison of neutrophil elastase detection findings at asthma attack; (5) comparison of genomic data; (6) determination of the predictors of clinical and inflammatory responses to prednisolone treatment; and (7) inhaler adherence data, which will be obtained from prescription refill records at asthma attack visit.

### Sample Size Requirements

The clinical and inflammatory effects of prednisolone for asthma attacks in patients treated with asthma mAbs are unknown. Therefore, an important aim of this observational study is to understand the proportions and SDs of key measurements related to these effects. These will help determine if a future randomized placebo-controlled study of prednisolone treatment for attacks in people treated with asthma mAb therapy should be FeNO-directed.

Because there are no published data on treatment response to prednisolone for asthma attacks in this population, we based our hypothesis and sample size estimates on our clinical experiences with attacks that we assessed and treated locally over recent years. We estimated the proportion of treatment failure at 28 days to be 0.05 in the high-FeNO group and 0.35 in the low-FeNO group. Further, based on the MEX study, in which roughly half of the asthma attacks among patients on mAbs were high-FeNO attacks and the other half were low-FeNO attacks, we estimated an average treatment failure proportion of 0.2 for our sample. This proportion is consistent with published systematic review data on the treatment failure at 28 days after an asthma attack (ie, among patients not on mAb therapy) [13].

Using a 2-sided α of .05, a β of .8, an equal high-FeNO and low-FeNO group ratio, and a 10% dropout rate, the total sample size required to detect the estimated proportions of treatment failure described above was calculated to be 60 asthma attacks. Additionally, 60 asthma attacks are also adequate for detecting a clinically meaningful difference in the change in FEV_1_ from day 0 to day 7 between the high-FeNO and low-FeNO groups (mean 0.4 vs mean 0, SD 0.5 L). Further, 60 attacks can be used detect a clinically meaningful difference in the change in VAS symptom score from day 0 to day 7 between the high-FeNO and low-FeNO groups (mean 15 vs mean 0, SD 17 mm).

### Statistical Analysis Plan

#### Overview

Descriptive statistics will be used to describe variables. For continuous variables, the differences in the means and the corresponding 95% CIs will be reported between the high-FeNO and low-FeNO groups and for the overall sample. For categorical variables, chi-square tests will be used for comparing treatment groups if the variables are normally distributed. If a variable is not normally distributed, a nonparametric test will be used for the analysis.

#### Primary Analysis

In our study, treatment failure will be defined as a need to start oral prednisolone or antibiotics or an unscheduled health care visit for asthma.

The comparison of proportions of treatment failure at day 28 between the high-FeNO and low-FeNO groups will be conducted with a Fisher exact test. The comparison of the time to treatment failure between the high-FeNO and low-FeNO groups will be presented with a Kaplan-Meier analysis. Additionally, we will construct a Cox proportional-hazards model to compare the time to treatment failure between high-FeNO and low-FeNO groups based on the following two interactions: (1) specific mAb treatment and (2) the use of antibiotics within the 2 weeks preceding the attack visit (inclusive of the attack visit day). We will construct a forest plot to assess potential predictors of treatment failure among the high-FeNO and low-FeNO groups.

There is no consensus for a low-FeNO versus high-FeNO attack threshold regarding response to prednisolone. For the purposes of the primary statistical analysis, we prespecified 25 parts per billion (ppb) as the main threshold to define low-FeNO and high-FeNO attacks. This threshold is based on sputum eosinophil data from the MEX study [10].

Any missing treatment failure outcome data will be dealt with via nonresponder imputation.

#### Secondary Analysis

We will analyze treatment failure by using a threshold of 50 ppb to define low-FeNO and high-FeNO attacks. This is based on data showing that an FeNO level of ≥50 ppb (high FeNO) is associated with a high risk of future asthma attacks for any patient with asthma [14].

We will use linear mixed-effects models to compare continuous data from the three secondary outcomes, across different time points, between high-FeNO and low-FeNO attacks. The random effect will be the participants. The fixed effects will be (1) specific mAb treatment, (2) the use of antibiotics within the 2 weeks preceding the attack visit (inclusive of the attack visit day), (3) the number of asthma attacks in the 12 months prior to the attack visit, and (4) percent predicted FEV_1_ at the attack visit.

Any missing secondary outcome data will be dealt with in the linear mixed-effects model by using the missing at random assumption.

The prediction of clinical responses to prednisolone will be performed via the generation of receiver operator characteristic curves and correlation coefficients, cluster analysis, and regression models.

#### Exploratory Analysis

We will run paired statistical tests and regression models to analyze the exploratory outcomes.

## Results

Recruitment started in September 2022. The target number of asthma attacks is expected to be reached by December 2023. Data analysis and publication will take place in 2024. Results will be published in peer-reviewed journals.

## Discussion

Our observational study aims to explore whether the presence of type 2 inflammation, as measured based on FeNO, predicts response to oral prednisolone for an asthma attack in an mAb-treated person. The study is novel because this response has not yet been assessed in a structured way before and after prednisolone treatment. We will measure response by using treatment failure, as this is a clinically important outcome for both people with asthma and health care professionals. It represents disruption to a person’s life, as well as health care utilization. Additionally, it is equivalent to the relapse outcome measured in the randomized placebo-controlled trials of OCSs in the 1980s and 1990s [4]. Other important outcomes will include symptom recovery, which is important to patients, and lung function, which captures a physiological change. Exploratory outcomes will include nasal, sputum, and blood inflammatory markers, which will be measured by using immunoassays and transcriptomics to assess whether high-FeNO attacks display evidence of ongoing pathways of inflammation not targeted by the mAb treatment.

There are several goals in our observational study. First, we expect to see a better clinical response in high-FeNO asthma attacks than in low-FeNO asthma attacks among people on mAb therapy. This is based on evidence from COPD exacerbations, wherein biomarker evidence of inflammation predicts response to OCSs—an anti-inflammatory treatment [8]. These data will help inform whether a future randomized controlled trial of prednisolone treatment for nonsevere attacks in patients treated with asthma mAbs should be FeNO-directed. This future trial is important because OCSs have significant side effects; therefore, a precision medicine approach is preferable to the existing one-size-fits-all approach. Second, we will start to explore the question about whether high-FeNO attacks have different underlying pathophysiological processes that are still steroid-responsive. This will provide novel data to the field, and such data are important, as they will help with the development of biomarkers that can predict whether certain people will respond better to a particular mAb therapy.

The main limitation of the study is that it is observational in nature, with no randomization, blinding, or placebo control. It is therefore subject to selection, reporting, and experimenter biases. We have tried to minimize these with our inclusion and exclusion criteria and by controlling for confounders in the statistical analyses. Ultimately, our study is hypothesis-generating research and is pilot work on which a larger, definitive trial will be based.

With our study, we hope to continue precision medicine research in asthma and begin to challenge the dogma that OCSs are effective in all asthma attacks.

## Abbreviations

BOOST: Breakthrough Asthma Attacks Treated With Oral Steroids
COPD: chronic obstructive pulmonary disease
FeNO: fractional exhaled nitric oxide
FEV_1_: forced expiratory volume in 1 second
ICS: inhaled corticosteroid
IL: interleukin
mAb: monoclonal antibody
OCS: oral corticosteroid
ppb: parts per billion
VAS: visual analogue scale

## References

[1] Martin MJ, Beasley R, Harrison TW (2020). Towards a personalised treatment approach for asthma attacks. Thorax.

[2] Pavord ID, Afzalnia S, Menzies-Gow A, Heaney LG (2017). The current and future role of biomarkers in type 2 cytokine-mediated asthma management. Clin Exp Allergy.

[3] Settipane RA, Kreindler JL, Chung Y, Tkacz J (2019). Evaluating direct costs and productivity losses of patients with asthma receiving GINA 4/5 therapy in the United States. Ann Allergy Asthma Immunol.

[4] Rowe BH, Spooner CH, Ducharme FM, Bretzlaff JA, Bota GW (2007). Corticosteroids for preventing relapse following acute exacerbations of asthma. Cochrane Database Syst Rev.

[5] Price DB, Trudo F, Voorham J, Xu X, Kerkhof M, Jie JLZ, Tran TN (2018). Adverse outcomes from initiation of systemic corticosteroids for asthma: long-term observational study. J Asthma Allergy.

[6] Brusselle GG, Koppelman GH (2022). Biologic therapies for severe asthma. N Engl J Med.

[7] Menzies-Gow A, Bafadhel M, Busse WW, Casale TB, Kocks JWH, Pavord ID, Szefler SJ, Woodruff PG, de Giorgio-Miller A, Trudo F, Fageras M, Ambrose CS (2020). An expert consensus framework for asthma remission as a treatment goal. J Allergy Clin Immunol.

[8] Bafadhel M, McKenna S, Terry S, Mistry V, Pancholi M, Venge P, Lomas DA, Barer MR, Johnston SL, Pavord ID, Brightling CE (2012). Blood eosinophils to direct corticosteroid treatment of exacerbations of chronic obstructive pulmonary disease: a randomized placebo-controlled trial. Am J Respir Crit Care Med.

[9] Ramakrishnan S, Jeffers H, Langford-Wiley B, Davies J, Mahdi M, A'Court C, Binnian I, Bright S, Cartwright S, Fox R, Russell RE, Bafadhel M (2022). Point of care blood eosinophil guided oral prednisolone for COPD exacerbations: a multi-centre double blind randomised controlled trial (The STARR2 trial). Eur Respir J.

[10] McDowell PJ, Diver S, Yang F, Borg C, Busby J, Brown V, Shrimanker R, Cox C, Brightling CE, Chaudhuri R, Pavord ID, Heaney LG, Medical Research Council: Refractory Asthma Stratification Programme (RASP-UK Consortium) (2021). The inflammatory profile of exacerbations in patients with severe refractory eosinophilic asthma receiving mepolizumab (the MEX study): a prospective observational study. Lancet Respir Med.

[11] Smith S, Gillespie L, Heaney L, Brightling C, Mcconnachie A, Siddiqui S, Mistry V, Brown V, Jackson D, Lee N, Fowler S, Mansur A, Burhan H, Saralaya D, Kurukulaaratchy R, Nasser S, Pfeffer P, Brown T, Lordan J, Pavord I, Chaudhuri R (2021). Adapting asthma clinical trials in response to the COVID-19 pandemic - the BenRex study design. Eur Respir J.

[12] Couillard S, Pavord ID, Heaney LG, Petousi N, Hinks TSC (2022). Sub-stratification of type-2 high airway disease for therapeutic decision-making: A 'bomb' (blood eosinophils) meets 'magnet' (FeNO) framework. Respirology.

[13] Hill J, Arrotta N, Villa-Roel C, Dennett L, Rowe BH (2017). Factors associated with relapse in adult patients discharged from the emergency department following acute asthma: a systematic review. BMJ Open Respir Res.

[14] Couillard S, Laugerud A, Jabeen M, Ramakrishnan S, Melhorn J, Hinks T, Pavord I (2022). Derivation of a prototype asthma attack risk scale centred on blood eosinophils and exhaled nitric oxide. Thorax.

